# Emotional Impact of Patient Death and Associated Psychological Distress in Cardiac Physicians

**DOI:** 10.1016/j.jacadv.2026.102861

**Published:** 2026-06-17

**Authors:** Thibaud Damy, Sophie Provenchère, Frédéric Pochard, Rebecca Dickason, Erwan Flécher

**Affiliations:** a French Referral Centre for Cardiac Amyloidosis, Cardiology Department, Henri-Mondor, GRC Amyloid Research Institute, Amyloidosis Mondor Network, Henri-Mondor Teaching Hospital, Creteil, France; b Clinical Epidemiology and Ageing (CEpiA) Geriatrics, Primary Care and Public Health and, Université Paris Est Creteil, INSERM, IMRB, Créteil, France; c Groupe Insuffisance Cardiaque et Cardiomyopathies (GICC) de la Société Française de Cardiologie (SFC), Paris, France; d Department of Anesthesiology and Intensive Care, Hôpital Bichat, Paris, France; e Anesthésie Réanimation, Cœur Thorax Vaisseaux (ACORTHOVA), Paris, France; f Famirea Medical Intensive Care Unit, APHP – CHU Saint Louis, Paris, France; g IGR-IAE Graduate School of Management, University of Rennes, Rennes, France; h CNRS, CREM – UMR 6211, Rennes, France; i Cardiothoracic Surgical Department, CHU Pontchaillou, Rennes, France; j Société Française de Chirurgie Cardio-thoracique, (SFCTV), Paris, France

**Keywords:** anxiety, burnout, cardiac physicians, death, mental health symptoms, post-traumatic stress disorder

## Abstract

**Background:**

Despite the frequent occurrence of patient death in cardiac practice, its emotional impact on cardiac physicians (CPs) remains poorly explored.

**Objectives:**

The objective of the study was to assess the Emotional Impact of Patient Death (EIPD) and examine its association with symptoms of psychological distress, including anxiety, depression, post-traumatic stress disorder, and burnout. A secondary objective was to identify risk factors of high emotional impact.

**Methods:**

Cross-sectional survey of French CPs, using EIPD self-reported 10-point Likert scale, the Hospital Anxiety and Depression Scale, the Impact of Event Scale-Revised, and the Maslach Burnout Inventory.

**Results:**

Among 6,080 invited physicians, 747 CPs completed the survey (response rate 12.2%; mean age 44.5 ± 12.3 years; 43% women; 75% cardiologists, 12% anesthesiologists, 8% surgeons, and 5% pediatric cardiologists). CPs were divided into EIPD quartiles; Q4 (≥8/10) comprised 201 (26%) respondents. Higher EIPD was associated with a greater psychological distress: Q4 participants reported symptoms of anxiety (n = 104, 52%), depression (n = 85, 42%), post-traumatic stress disorder (n = 121, 60%), and severe burnout (n = 103, 51%). This group also reported more frequent conflicts with colleagues and higher use of psychotropic or addictive substances. In multivariable analysis, female sex, cardiothoracic surgical specialty, and a history of personal trauma were independently associated with high EIPD (Q3–Q4 vs Q1–Q2).

**Conclusions:**

The emotional impact of patient death is significantly associated with symptoms of psychological distress among CPs, highlighting an under-recognized dimension of physician well-being that requires targeted institutional support and training.

The rising prevalence of symptoms of psychological distress among cardiac physicians (CPs: cardiologists, cardiothoracic surgeons, cardiovascular anesthesiologists, intensive care specialists, and pediatric cardiologists) is increasingly recognized, with 1 in four cardiologists reporting mental health issues in a recent global survey conducted by the American College of Cardiology among almost 6,000 physicians.^1^^,^^2^ Reported conditions include psychological distress such as anxiety, irritability, anger, depression, and post-traumatic stress disorder (PTSD).^1^^,^^3^^,^^4^ These difficulties are associated with an alarming rate of burnout among physicians in general and among cardiologists.^5^^,^^6^ Indeed, burnout has been reported among CPs.^7^^,^^8^^,^^9^ This consistently high burnout rate is mostly driven by health care systems and organizational issues.^10^

CPs work in highly demanding specialties, with complex, multidimensional clinical roles involving life-altering decisions concerning the heart. These decisions may at times involve end-of-life situations (eg whether to continue resuscitation maneuvers), not only in acute situations but also after complex technical procedures and during the follow-up of chronic heart disease. The emotional toll of making such decisions places these physicians at risk of compassion fatigue.^11^ In addition, many physicians have roles in training, leadership, team management, or administrative and research activities, further increasing their workload in terms of energy demands, time management, and multitasking.^12^

In other medical specialties, psychological distress due to patient deaths, including compassion fatigue,^13^ grief,^14^ anxiety or PTSD,^15^^,^^16^ has been reported. Conversely, little is known about the psychological impact of patient death among CPs, particularly in stressful situations that may be compounded by unpredictable external pressures inherent to this specialty. Qualitative research has highlighted that physicians can also experience strong and lasting emotional reactions in response to certain patient deaths and underscores the complexity of balancing personal and professional responses.^17^^,^^18^

Burnout among CPs has significant personal and professional consequences and is associated with suboptimal health care outcomes for patients.^2^^,^^18^^,^^19^

This study investigates the relationship between these mental health symptoms and the emotional impact of patient death as well as factors related to patient’s characteristics and death circumstances.

## Methods

### Study design, setting, and participants

This multicenter, cross-sectional survey of hospital-based CPs was conducted between July and September 2021 in France. The survey was anonymous.

All cardiologists, cardiac surgeons, cardiac anesthesiologists, and intensive care physicians registered with the French Society of Cardiology (Société Française de Cardiology) and French Society of Cardiac and Cardiovascular Surgery (Societé Française de chirurgie thoracique et cardiovasculaire), the French Society of Cardiothoracic Anesthesia, and the French multidisciplinary research group on families in intensive care group mailing list were invited by email to complete the confidential, online, and self-reported questionnaire. Physicians in training were defined as residents and fellows, whereas senior physicians correspond to attending physicians.

The French multidisciplinary research group on families in intensive care group is an independent, multidisciplinary French research group whose research focuses on the experiences of families with patients in intensive care units. The invitation email described the study objectives, confirmed anonymity, and included a link to the survey. The initial email was sent in July 2021, followed by a reminder in September 2021. According to French legislation, ethics approval is not required for anonymous surveys not involving patients. All participants provided informed consent before accessing the questionnaire.

### Primary outcome

The primary objective was to measure the Emotional Impact of Patient Death (EIPD), assessed using a self-reported 10-point Likert scale ranging from 1 (no emotional impact) to 10 (maximum emotional impact), designed to capture the immediate subjective emotional intensity experienced at the time of patient death. As this measure was intentionally designed as a single-item global assessment, internal consistency metrics such as Cronbach’s alpha, which require multiple items assessing the same construct, are not applicable.

The EIPD was created specifically for the purposes of this study by a multidisciplinary working group including a psychiatrist, a cardiac surgeon, an intensive care physician, a sociologist, and a cardiologist. The group met 16 times to review the literature and define relevant questionnaire items. The final questionnaire was pilot-tested in 10 CPs from different specialties to assess readability and comprehension. No modifications were required, and pilot data were not included in the final analysis.

### Secondary outcome

Secondary objectives were to examine the association between EIPD and psychological distress, including symptoms of anxiety and depression, PTSD-related symptoms and burnout, and to identify factors associated with EIPD.

### Survey questionnaire and instruments

Participants completed the online self-report questionnaire in French. An English version is provided in the Supplemental Material. The questionnaire consisted of 3 sections.

#### Demographics and professional characteristics

This section collected data on age, gender, family situation, professional status, type of practice (private practice, public or private hospital), and years of work experience.

Professional activity was characterized by the mean number of hours worked per week, number of on-call shifts per week, and number of days without a full week of uninterrupted leave. Relationships with patients and colleagues were assessed using 10-point Likert scales (1 = poor, 10 = excellent).

#### Experience of patient death and EIPD

This section assessed the emotional impact of patient death using the EIPD (10-point Likert scale; 1 = no impact, 10 = maximum impact), based on the question: “Do you think that the deaths of your patients have an emotional/affective impact on you?”

Additional data included the number of patient deaths experienced in the past month and year, characteristics of death (expected vs unexpected, procedural context, and COVID-19 involvement), palliative care decisions, and perceived support from family, colleagues, and institutions.

Participants were also asked about coping behaviors, including substance use, psychotropic medication use, and psychological support.

#### Psychological assessment tools

The third section included 3 validated instruments.

• The Hospital Anxiety and Depression Scale (HADS) was used to assess symptoms of anxiety and depression, originally developed for medically ill populations.^20^ In this study, it was used as a screening tool and not for diagnostic purposes. Each subscale (anxiety and depression) ranges from 0 to 21, with scores of 0 to 7 considered normal, 8 to 10 borderline, and ≥11 indicative of probable disorder.
• The Impact of Event Scale-Revised (IES-R) was used to assess symptoms of PTSD.^21^

The IES-R is a self-report screening questionnaire the measures symptoms related to PTSD, due to the psychological impact of a traumatic event. It evaluates 3 dimensions, namely intrusion, avoidance, and hyperarousal. Respondents rate symptoms experienced in the past 7 days related to a specific traumatic event. There are 22 items and responses are on a 5-point Likert scale, for a maximum score of 88 points. Scores of 33 to 36 are suggestive of a moderate impact, and scores of 37 or higher are indicative of a severe impact (likely symptoms of PTSD).

• The Maslach Burnout Inventory (MBI) was used to assess burnout.^22^ MBI assesses possible burnout symptoms across 3 core dimensions, namely emotional exhaustion, depersonalization, and personal accomplishment. Responses are on a 7-point Likert scale. The combination of high emotional exhaustion, high depersonalization, and low personal accomplishment scores is suggestive of burnout.

### Statistical analysis

Respondent characteristics are summarized as mean ± SD or number (percentage) as appropriate. Likert scale results are expressed as median and IQR. Independent predictors of high EIPD (Q3–Q4 vs Q1–Q2) were identified using binary logistic regression. Variables significant in univariate analysis were entered into a multivariable model using conditional stepwise selection (entry *P* ≤ 0.20, removal *P* ≤ 0.10). Pairwise interaction terms between variables significant in univariate analysis were tested in unadjusted models before multivariable construction; no significant interactions were identified (all *P* > 0.10). Continuous variables for which log-linearity was not confirmed were categorized according to the median or IQR. A mixed model with center as a random effect was used as the final preplanned model. Missing data were handled by complete case analysis (n = 747; completion rate 80.8%); item-level missing data were minimal as the survey platform required completion of each section before proceeding. No adjustments for multiple comparisons were performed. As a sensitivity analysis, EIPD was also treated as a continuous outcome in a linear regression model including the same predictors. All analyses were performed with SPSS 13.0 (SPSS Inc.). A *P* value <0.05 was considered significant. The study is reported in accordance with the Consensus-Based Checklist for Reporting of Survey Studies reporting guidelines.^23^

## Results

### Participant characteristics

**Figure 1:**
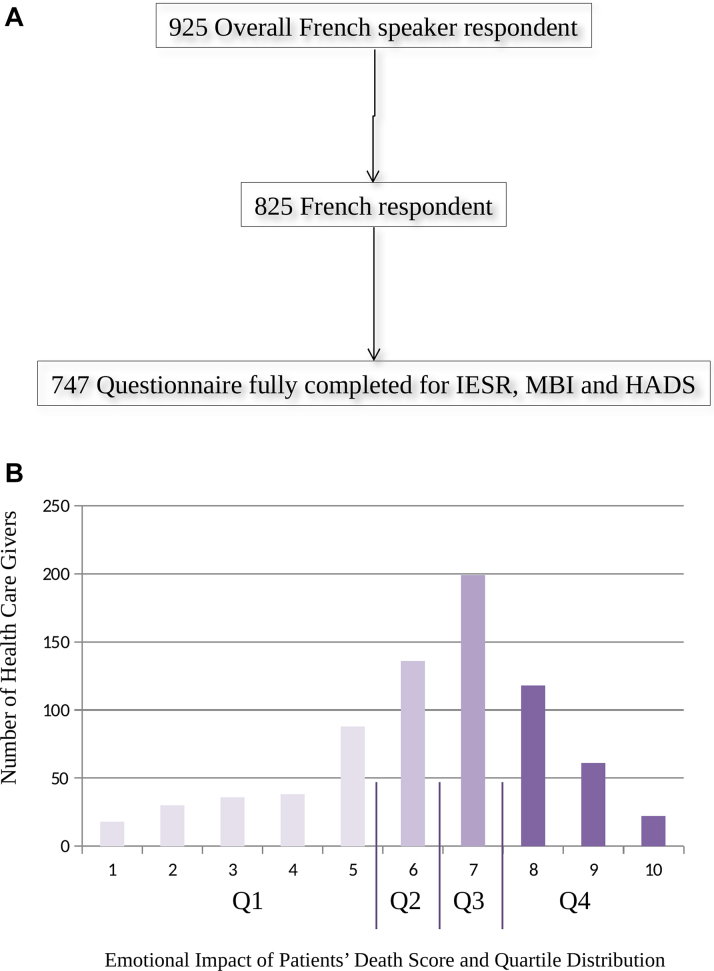
**Flowchart and Emotional Impact of Patient Death Scale Distribution by Quartiles** (A) Presents the flow chart of participant inclusion. (B) Shows the distribution of responses to the Emotional Impact of Patient Death (EIPD) scale, ranging from 1 to 10, with quartile boundaries indicated. The EIPD scale captures the subjective emotional impact experienced by cardiac physicians following patient death. Quartile 1 (Q1) corresponds to the lowest scores and Quartile 4 (Q4) to the highest. This distribution informed the quartile-based subgroup analyses used throughout the study. IES-R = Impact of Event Scale-Revised; HADS = Hospital Anxiety and Depression Scale; MBI = Maslach Burnout Inventory.

In total, 6,080 physicians were invited to participate, including cardiologists (n = 2,500), surgeons (n = 480), anesthesiologists (n = 1,500), pediatricians (n = 100), and physicians in training (n = 1,000) (Figure 1, Table 1).

**Table 1:** Demographic and Job Characteristics of the Overall Cohort and Comparisons Between the 3 Specialties

	All (N = 747)	Emotional Impact of Death on Cardiac Physicians				*P* Value
		Quartile 1 (n = 212)	Quartile 2 (n = 136)	Quartile 3 (n = 199)	Quartile 4 (n = 201)	
Age, y	44.5 ± 12.3	45.1 ± 12.6	42.3 ± 11.5	43.2 ± 12.1	46.7 ± 12.5	0.004
Females	322 (43.3)	69 (32.9)	58 (43.0)	95 (48.0)	100 (50.0)	0.002
Living arrangements						
Family	394 (52.7)	120 (56.9)	63 (46.3)	106 (53.3)	105 (52.2)	0.322
Couple	272 (36.4)	75 (35.5)	58 (42.6)	70 (35.2)	69 (34.3)	
Single	81 (10.8)	16 (7.6)	15 (11.0)	23 (11.6)	27 (13.4)	
Specialty						
Cardiologist for adults	562 (75.2)	154 (27.4)	102 (18.1)	154 (27.4)	152 (27.0)	0.001
Anesthetist or Intensive care	89 (11.9)	38 (42.7)	22 (24.7)	18 (20.2)	11 (12.4)	
Cardiovascular surgeon	62 (8.3)	12 (19.4)	8 (12.9)	18 (29.0)	24 (38.7)	
Cardiologist for children	34 (4.6)	7 (3.3)	4 (2.9)	9 (4.5)	14 (7.0)	
Cardiac physician position						
Physician in training (residents and fellows)	54 (7.2)	11 (20.4)	15 (27.8)	15 (27.8)	13 (24.1)	0.116
Senior registrar	95 (12.7)	34 (35.8)	12 (12.6)	30 (31.6)	19 (20)	
Physician in private practice	527 (70.5)	142 (26.9)	95 (18.0)	135 (25.6)	155 (29.4)	
Professor and senior lecturer	71 (9.5)	24 (33.8)	14 (19.7)	19 (9.5)	14 (7.0)	
Location of work						
Private practice	110 (14.7)	27 (24.5)	18 (16.4)	30 (27.3)	35 (31.8)	0.105
Public hospital	117 (15.7)	34 (29.1)	16 (13.7)	37 (31.6)	30 (25.6)	
University hospital	366 (43)	109 (29.8)	76 (20.8)	1°2 (27.9)	79 (21.6)	
Private hospital	96 (12.9)	25 (26.0)	16 (16.7)	18 (18.8)	37 (38.5)	
Mixed (private practice, public hospital)	23 (3.1)	12 (34.3)	6 (17.1)	5 (14.3)	12 (34.3)	
Mixed (private practice, private hospital)	35 (4.7)	4 (17.4)	4 (17.4)	7 (30.4)	8 (34.8)	

Values are mean ± SD or n (%).

Overall, 925 francophone physicians responded to the survey, of whom 825 (89.2%) were practicing in France. Among these, 747 (80.8%) completed the full three-part questionnaire and were included in the final analysis (Figure 1). The overall response rate based on analyzable questionnaires was therefore 12.2% (747/6,080). Cardiologists accounted approximately 75% the study population with the remainder consisting of surgeons, intensive care specialists, and pediatricians.

### Demographics characteristics

The mean age of respondents was 44.5 ± 12.3 years and 537 (70.5%) reported being a senior physician, 366 (43%) worked at a university hospital, and 342 (43%) were women. Concerning working conditions, the physicians reported working long hours and having long periods without uninterrupted leave. The mean number of hours worked per week was 55.0 ± 23.5 and the mean number of days worked without uninterrupted leave was 208.8 ± 316.1. CPs reported overall good relationships with colleagues (7.9 ± 1.5), although 248 (33%) nevertheless reported conflicting relationships within their teams (Table 2). Physicians reported having good relationships with patients (8.0 ± 1.3) and patients' families (7.4 ± 1.5). Physician salary was rated as unsatisfactory (4.9 ± 2.7) (Tables 1 and 2).

**Table 2:** Work Characteristics of the Overall Cohort and Comparison Between the Three Subspecialties

	All (N = 747)	Emotional Impact of Death on Cardiac Physicians				*P* Value
		Quartile 1 (n = 212)	Quartile 2 (n = 136)	Quartile 3 (n = 199)	Quartile 4 (n = 201)	
Number of years’ experience	19.3 ± 15.0	19.9 ± 15.6	16.7 ± 12.1	17.9 ± 14.2	22.0 ± 16.4	0.007
Hours worked per week	55.0 ± 23.5	53.7 ± 13.3	54.4 ± 11.6	55.1 ± 12.1	56.6 ± 40.4	0.554
Duration of daily commute (minutes)	44.2 ± 31.8	40.9 ± 25.4	42.3 ± 30.4	44.5 ± 29.3	48.7 ± 39.7	0.199
Number of night shifts/month	1.9 ± 2.1	2.0 ± 2.0	2.0 ± 1.9	1.8 ± 2.1	1.6 ± 2.2	0.045
Number of weekend shifts/month	3.8 ± 4.9	3.1 ± 3.6	3.2 ± 3.6	4.3 ± 4.9	4.3 ± 6.6	0.138
How long since a full week of holidays without working (days)	209 ± 316	198 ± 377	202 ± 294	219 ± 308	215 ± 266	0.02
Quality of relationship with Institution, 1 (very bad) to 10 (excellent)	5.0 ± 2.2	4.8 ± 2.4	5.3 ± 2.0	5.0 ± 2.2	5.2 ± 2.1	0.301
Do you work with other health care workers, yes	722 (96.7)	205 (28.4)	131 (18.1)	195 (27.0)	191 (26.5)	0.398
Quality of relationship with health care coworkers, 1 (very bad) to 10 (excellent)	7.9 ± 1.5	7.9 ± 1.5	7.7 ± 1.5	7.9 ± 1.5	8.2 ± 1.4	0.071
Conflictual relationship with coworkers						0.030
Yes	226 (30.3)	59 (28.0)	38 (18.0)	60 (28.4)	69 (32.7)	
No	506 (67.7)	149 (70.6)	91 (43.1)	139 (65.9)	127 (60.2)	
Not applicable	15 (2.0)	3 (1.4)	7 (3.3)	0 (0.0)	5 (2.4)	
Do you consider yourself to be paid adequately? (1-10)	4.9 ± 2.7	4.8 ± 2.6	4.9 ± 2.7	4.9 ± 2.7	5.2 ± 2.8	0.503
Quality of relationship with your patients? (1-10)	8.0 ± 1.3	7.8 ± 1.4	7.9 ± 1.2	8.0 ± 1.2	8.1 ± 1.3	0.084
Quality of relationship with the family of your patients (1-10)	7.4 ± 1.5	7.3 ± 1.5	7.2 ± 1.5	7.4 ± 1.5	7.6 ± 1.5	0.054

Values are mean ± SD or n (%).

### Characteristics of the patient deaths

The median number of deaths experienced in the previous year and month were respectively 10 (5-20) and 1 (1-2). Overall, 89 (12%) of respondents reported alcohol or cannabis consumption following a patient’s death, whereas 35 (5%) reported using psychotropic medication or consulting a psychologist and/or psychiatrist. Although physicians reported receiving support from colleagues, family, or friends, institutional support was perceived as insufficient (Table 3).

**Table 3:** Impact of Patient Death in the Overall Cohort and Comparison Between the Three Subspecialties

	All (N = 747)	Emotional Impact of Death on Cardiac Physicians				*P* Value
		Quartile 1 (n = 212)	Quartile 2 (n = 136)	Quartile 3 (n = 199)	Quartile 4 (n = 201)	
The death of your patients						
Do you think that your patients’ death has an impact on your feelings	6.3 ± 2.0	3.7 ± 1.4	6.0 ± 0.0	7.0 ± 0.0	8.5 ± 2.3	ND
Death of your patient						
Number of your patients who died in the last year?	10 (5-20) 16.3 ± 22.5	10 (5-20) 18.9 ± 30.3	10 (5-20) 18.4 ± 20.5	10 (5-20) 15.0 ± 15.6	10 (4-15) 13.4 ± 19.3	0.001
Number of your patients who died in the last month?	1 (1-2) 2.0 ± 2.6	2 (1-3) 2.2 ± 2.8	2 (1-3) 2.1 ± 2.2	1 (1-3) 1.9 ± 2.1	1 (0-2) 1.8 ± 3.0	0.003
Prevalence of unexpected deaths (acute death) median IQR	10 (2; 25)	10 (1; 25)	10 (1; 25)	10 (3; 30)	10 (1; 25)	0.213
How many palliative care decisions were you involved in over the last month (1 to more than 10)	2.6 ± 2.0	2.5 ± 2.0	2.8 ± 2.1	2.8 ± 2.0	2.4 ± 1.8	0.190
Could you estimate your emotional impact when faced with…						
Death occurring after a long follow-up	7.0 ± 2.3	6.0 ± 2.5	7.2 ± 1.9	7.1 ± 2.1	7.7 ± 2.3	0.0001
When you identify with the patient	5.8 ± 2.8	5.2 ± 2.9	5.9 ± 2.6	5.9 ± 2.7	6.4 ± 2.8	0.0001
Death of a young patient	8.1 ± 1.9	7.5 ± 2.0	8.1 ± 1.7	8.4 ± 1.7	8.4 ± 2.1	0.0001
Death due to a cardiovascular cause	5.1 ± 2.9	3.8 ± 2.7	5.1 ± 2.7	5.2 ± 2.7	6.4 ± 2.8	0.0001
Unexpected death (sudden)	7.6 ± 2.1	6.6 ± 2.3	7.7 ± 1.7	7.7 ± 1.8	8.4 ± 1.8	0.0001
Death occurring during an intervention	7.9 ± 2.2	7.1 ± 2.6	7.8 ± 2.2	8.0 ± 2.0	8.7 ± 1.8	0.0001
Death occurring after an intervention	7.4 ± 2.1	6.5 ± 2.3	7.1 ± 2.0	7.7 ± 1.8	8.2 ± 1.8	0.0001
Following the death of your patients did you…						
Take psychotropic drug(s)						0.002
Yes	35 (5)	4 (1.9)	4 (2.9)	7 (3.5)	20 (10)	
No	707 (94.6)	207 (98.1)	131 (06.3)	189 (95.0)	180 (89.6)	
I prefer not to reply	5 (0.7)	0 (0)	1 (0.7)	3 (1.5)	1 (0.5)	
Consult a psychologist or psychiatrist,						0.0001
Yes	35 (4.7)	4 (1.9)	1 (0.7)	6 (3.0)	24 (11.9)	
No	176 (87.6)	206 (97.6)	134 (98.5)	192 (96.5)	175 (87.6)	
I prefer not to reply	4 (0.5)	1 (0.5)	1 (0.7)	1 (0.5)	1 (0.5)	
Take substances (alcohol, cannabis), yes						0.002
Yes	89 (11.9)	13 (6.2)	11 (8.1)	30 (15.1)	35 (17.4)	
No	651 (87.1)	198 (30.4)	124 (19.0)	167 (83.9)	162 (80.6)	
I prefer not to reply	4 (2.0)	0 (0)	1 (0.7)	2 (1.0)	4 (2.0)	
About you						
Do you have a traumatic history (death, accident, aggression)? 1 to 10	3.6 ± 2.9	2.7 ± 2.7	3.4 ± 2.7	3.9 ± 2.9	4.3 ± 3.1	0.0001
How do you estimate the quality of your relationship with your family and friends? 1 to 10	8.0 ± 1.8	8.1 ± 1.8	7.9 ± 1.6	8.2 ± 1.7	7.9 ± 1.8	0.271
Do you do sport every week? Yes vs no	514 (68.8)	155 (73.5)	86 (63.2)	134 (67.3)	139 (69.2)	0.230
Do you practice mindfulness or sophrology?						0.094
Yes	138 (18.5)	26 (18.8)	29 (21.0)	41 (29.7)	42 (20.9)	
No	606 (81.1)	185 (30.5)	107 (17.7)	157 (25.9)	157 (25.9)	
I do not want to reply	3 (0.4)	0 (0.0)	0 (0.0)	1 (0.5)	2 (1.0)	
Do you believe in a god?						0.079
Yes	235 (31.5)	52 (22.1)	40 (17.0)	63 (26.8)	80 (34.0)	
No	350 (46.9)	110 (31.4)	68 (19.4)	90 (25.7)	82 (23.4)	
I am wondering	147 (19.7)	42 (28.6)	25 (17.0)	42 (28.6)	38 (25.9)	
I prefer not to reply	15 (2.0)	7 (46.7)	3 (20.0)	4 (26.7)	1 (6.7)	
Do you go to religious services?						0.835
Yes	142 (19.0)	35 (16.6)	23 (16.9)	43 (21.6)	41 (20.4)	
No	587 (78.6)	171 (81.0)	11 (80.9)	152 (76.4)	154 (76.6)	
I prefer not to reply	18 (2.4)	5 (2.4)	3 (2.2)	4 (2.0)	6 (3.0)	
About the type of death						
Do you feel helped after the death of one of your patients?						
By your colleagues, 1 to 10	5.5 ± 2.8	5.5 ± 2.8	5.7 ± 2.8	5.6 ± 2.7	5.5 ± 3.0	0.871
By your institution, 1 to 10	1.5 ± 2.2	1.4 ± 2.2	1.6 ± 2.2	1.5 ± 2.1	1.5 ± 2.3	0.757
By your family or friends, 1 to 10	5.4 ± 3.1	5.0 ± 3.2	5.5 ± 2.9	5.6 ± 3.0	5.5 ± 3.2	0.166
How many symptomatic COVID-19 patients did you treat in acute or chronic phases	10 (2; 30)	10 (2; 30)	15 (2; 30)	15 (5; 35)	10 (24; 41)	0.060
Do you think that there is sufficient teaching about the end of life or death during medical school? Yes vs No and I don’t know	37 (5.0)	15 (7.1)	12 (8.8)	8 (4.0)	2 (1.0)	0.007

Values are mean ± SD, median (IQR), or n (%).

ND = Not Determined.

### Emotional impact of patient death

**Figure 2:**
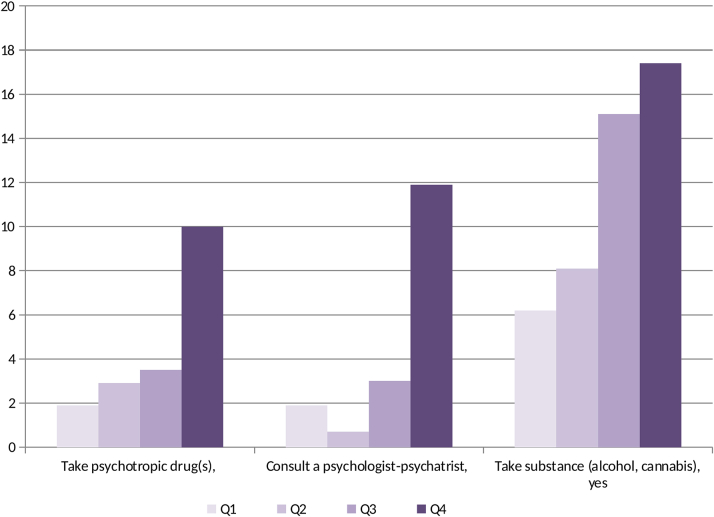
**Psychotropic Use, Substance Use, and Psychological Support by Emotional Impact of Patient Death Quartile** Percentage of physicians reporting psychotropic drug use, psychological consultation, and substance use, stratified by quartile of the Emotional Impact of Patient Death (EIPD) scale. Higher EIPD quartiles were associated with greater use of psychotropic medications and substances, whereas recourse to psychological support remained low across all groups, including among physicians with the highest emotional burden.

**Figure 3:**
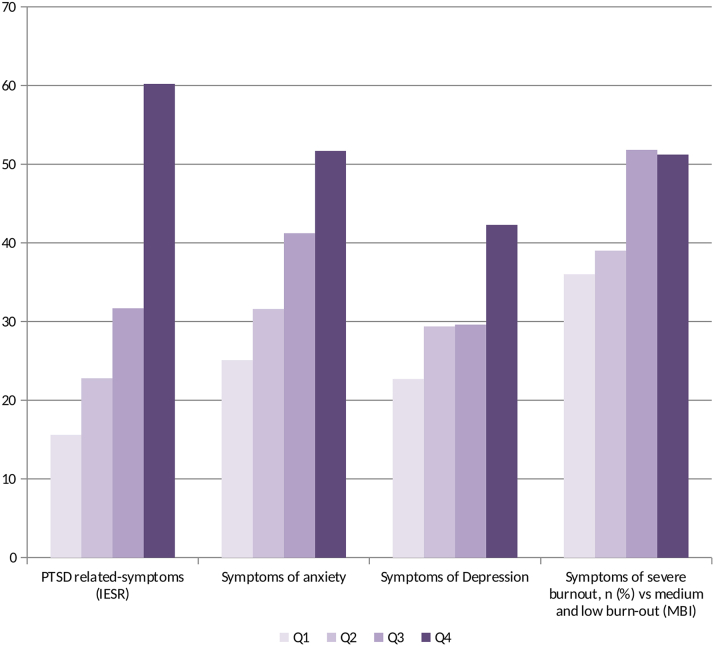
**Psychological Distress Prevalence Across Emotional Impact of Patient Death Quartiles in Cardiac Physicians** Prevalence of post-traumatic stress disorder (PTSD)-related symptoms, anxiety, depression, and burnout across quartiles of the Emotional Impact of Patient Death (EIPD) scale. Screening thresholds were defined using validated instruments: the IES-R for PTSD, the HADS for anxiety and depression, and the MBI for burnout. The proportion of physicians exceeding screening thresholds increased progressively with EIPD quartile. Abbreviations as in Figure 1.

The distribution of EIPD scores is shown in Figure 1 (Figures 1B, 2, and 3, Table 3, Table 4, Table 5). The median EIPD score was 6.3 (IQR: 5-8) (Table 3). Overall, 536 respondents (83%) reported an EIPD score ≥5, and 26% reported a score ≥8 (Central Illustration). Higher EIPD was associated with several factors, including a history of PTSD and increased workplace conflict. Among physicians with EIPD ≥8, 60 (26.5%) reported conflicts with colleagues compared with 38 (16.8%) among those with lower scores. The frequency of psychotropic medication use, substance use, and psychological support across EIPD quartiles is presented in Figure 2. Among the 201 physicians (26%) with EIPD ≥8, 35 (17.4%) reported substance use, 20 (10%) reported psychotropic medication use and only 24 (12%) sought psychological support following a patient's death.

**Table 4:** Death Emotional Impact on Physician Symptoms of Anxiety, Depression, PTSD-Related Symptoms, and Burnout

	All (N = 747)	Emotional Impact of Death on Physician				*P* Value
		Quartile 1 (n = 212)	Quartile 2 (n = 136)	Quartile 3 (n = 199)	Quartile 4 (n = 201)	
PTSD related-symptoms (IES-R)	248 (33.2)	33 (15.6)	31 (22.8)	63 (31.7)	121 (60.2)	0.0001
Intrusion	8 (4-14)	4 (2-8)	6 (4-11)	8 (5-14)	14 (9-21)	0.0001
Avoidance	5 (2-10)	3 (1-7)	4 (2-9)	5 (2-10)	9 (5-13)	0.0001
Hyperarousal	3 (1-6)	2 (0-3)	3 (1-6)	4 (2-7)	6 (3-10)	0.0001
Symptoms of anxiety	282 (37.8)	53 (25.1)	43 (31.6)	82 (41.2)	104 (51.7)	0.0001
Symptoms of depression	232 (31.1)	48 (22.7)	40 (29.4)	59 (29.6)	85 (42.3)	0.0001
Symptoms of severe burnout, medium and low burnout (MBI)	335 (44.8)	76 (36.0)	53 (39.0)	103 (51.8)	103 (51.2)	0.001
Exhaustion	20 (12-29)	15 (9-24)	17 (11-28)	22 (14-29)	24 (16-33)	0.0001
Depersonalization	7 (4-13)	8 (4-14)	8 (4-12)	7 (4-13)	6 (3-12)	0.243
Personal achievement	40 (35-44)	39 (35-44)	40 (35-43)	39 (35-44)	40 (34-44)	0.877

Values are n (%) or median (IQR).

IES-R = Impact of Event Scale-Revised; MBI = Maslach Burnout Inventory; PTSD = post-traumatic stress disorder; other abbreviation as in Table 1.

**Table 5:** Multivariate Analysis of Q3-Q4 vs Q1-Q2 of Death Emotional Impact

Binary Logistic Regression Model Variable	Univariate Analysis				Multivariate Analysis			
	Wald	OR	IC	*P* Value	Wald	OR	IC	*P* Value
Number of year at this job, year	1.279	0.993	0.981-1.005	0.258				
Work hour in a week, hour	1.228	1.008	0.994-1.024	0.268				
Last time of full week of holidays, day	0.066	1.000	1.000-1.001	0.798				
Number of death last year, n	0.591	0.997	0.990-1.004	0.442				
Personal post-traumatic disorders (1-10)	17.737	1.146	1.075-1.220	0.000	18,592	1,145	1,077-1,218	
Gender, women vs men	9.001	1.772	1.219-2.575	0.003	10,112	1,780	1,248-2,539	0.001
Specialty type (ref: anesthesiologist)	7.764			0.051	9,170			0.027
Cardiothoracic surgeon	6.371	2.908	1.269-6.662	0.012	7,912	3,131	1,414-6,936	0.005
Cardiopediatrician	2.790	2.340	0.863-6.348	0.095	3,025	2,363	0,897-6,230	0.082
Cardiologist	4.306	1.707	1.030-2.829	0.038	3,956	1,637	1,007-2,660	0.047
HADS anxiety, >7 vs <7	0.380	0.871	0.560-1.353	0.538				
HADS depression, >7 vs <7	0.294	0.887	0.575-1.369	0.588				
PTSD-related symptoms (IES-R)	17.078	2.636	1.665-4.175	0.000	25,635	2,973	1,950-4,533	
Symptoms of severe burnout, n (%) vs medium and low burnout (MBI)	0.559	0.859	0.577-1.279	0.454				
Conflict at work with a health care giver, yes vs no or NA	0.062	1.051	0.709-1.560	0.803				
Death course taught during medical school	0.642	1.349	0.648-2.808	0.423				

HADS = Hospital Anxiety and Depression Scale; NA = Not applicable; other abbreviations as in Tables 1 and 4.

**Central Illustration:**
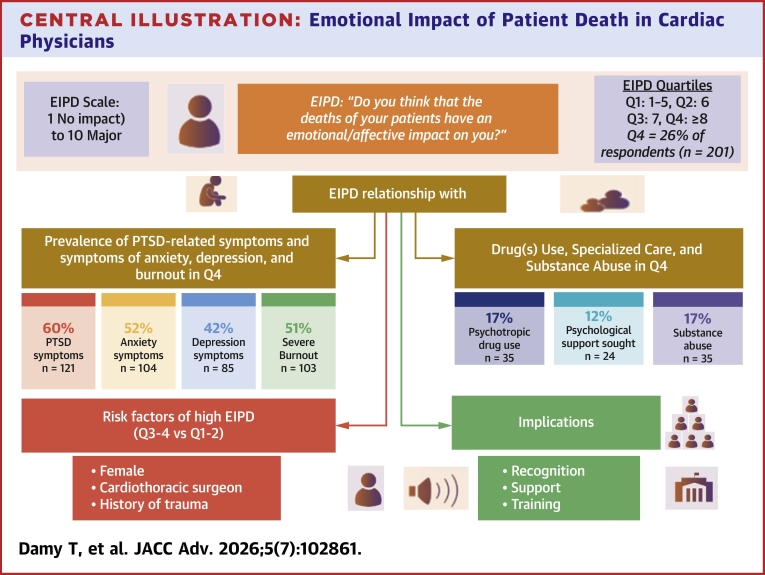
**Emotional Impact of Patient Death in Cardiac Physicians** This illustration summarizes the key findings of the study. The EIPD scale (1-10) assessed the subjective emotional impact of patient death. Among physicians in Q4 (EIPD ≥8, n = 201, 26%), 60% met screening thresholds for PTSD-related symptoms, 52% for anxiety, 42% for depression, and 51% for severe burnout. In the same group, 17% reported substance use and 17% psychotropic drug use, whereas only 12% sought psychological support. Female gender, cardiothoracic surgical specialty, and personal history of trauma were independently associated with high EIPD. EIPD = Emotional Impact of Patient Death; PTSD = post-traumatic stress disorder.

### Psychological outcomes

Table 4 presents HADS, IES-R, and MBI scores across EIPD quartiles (Table 4 and Figure 3). Overall, 248 (33%) physicians reported PTSD-related symptoms, 282 (38%) symptoms of anxiety, 232 (31%) symptoms of depression, and 335 (45%) burnout. Among the 201 (26%) physicians who had EIPD ≥8, 60.2% had symptoms of PTSD, 51.7% anxiety, 42.3% depression, and 51.2% severe burnout (Figure 3). In an exploratory analysis, EIPD scores were significantly higher among physicians with PTSD-related symptoms (mean 7.32 vs 5.78; *P* = 0.004) and severe burnout (mean 6.63 vs 6.02; *P* = 0.002), whereas no significant difference was observed for symptoms of anxiety (*P* = 0.068) or depression (*P* = 0.275), suggesting that high EIPD reflects a specific emotional reactivity to patient death rather than general psychological distress (Supplemental Figure 1).

### Factors associated with high emotional impact

In multivariable analysis, female gender, cardiothoracic surgical specialty, and a history of personal trauma were independently associated with high EIPD (Q3–Q4 vs Q1–Q2) (Tables 5, Supplemental Tables 1 and 2). Sensitivity analysis with EIPD as a continuous outcome yielded consistent results. In an exploratory comparison, cardiothoracic surgeons (n = 62) reported significantly higher EIPD scores than nonsurgeon physicians (n = 685) (6.90 vs 6.24, *P* = 0.017), despite experiencing fewer patient deaths per year (*P* = 0.001).

## Discussion

This study highlights the significant emotional impact of patient death on the psychological well-being of CPs. To the best of our knowledge, this is the first study to assess the relationship between the emotional impact of patient death and psychological distress in CPs, and to identify associated risk factors (including type of death and type physician specialty).

### Cardiac physician risk factors for high emotional impact

We found that female gender was associated with a higher emotional impact of patient deaths (OR: 1.78; 95% CI: 1.248-2.539). This finding aligns with previous reports among intensive care specialists, suggesting a higher likelihood of burnout symptoms among female physicians. Although the underlying mechanisms remain unclear, gender-related factors, including bias and discrimination, may contribute.^6^^,^^9^^,^^24^^,^^25^ A recent meta-analysis also reported higher suicide rates among female physicians compared with their male counterparts.^26^ Nevertheless, literature on gender differences is heterogeneous, and findings across studies remain inconsistent.

A personal history of trauma was also independently associated with higher emotional impact (OR: 1.145; 95% CI: 1.077-1.218). This relationship likely reflects complex and bidirectional mechanisms. Physicians with prior traumatic experiences may exhibit increased emotional reactivity when confronted with patient death. Conversely, repeated exposure to patient death may reactivate or exacerbate pre-existing traumatic memories or symptoms.^25^ Earlier exposure to violence during the physician’s childhood may also impact their adult response to stress and disrupt their emotional regulation processes.^27^

Cardiothoracic surgeons were at a higher risk of elevated emotional impact (OR: 3.131; 95% CI: 1.414-6.96). This may relate to the specific nature of surgical practice, which involves direct physical and visual engagement with their patients during surgery, a high level of perceived responsibility for outcomes, and a close temporal relationship between intervention and outcome, although this was not specifically explored in our study.

### Emotional and psychological consequences of patient death on cardiologists

Among the 201 (26%) physicians reporting a high emotional impact (EIPD ≥8), a substantial proportion met screening thresholds for PTSD-related symptoms (60.2%), symptoms of anxiety (51.7%), symptoms of depression (42.3%), and severe burnout (51.2%). As shown in Table 4, the increase in PTSD across EIPD quartiles was observed across all subdomains (intrusion, avoidance, and hyperarousal). Overall, 44.8% of respondents reported severe burnout.

Nevertheless, these symptoms of psychological distress have been reported in similar proportions among cardiologists^1^ and related specialties, and PTSD and depression frequently co-exist with burnout.^3^^,^^9^^,^^28^ Importantly, although these symptoms have a significant individual impact on physician well-being, respondents rated institutional support as low (median score 1.5/10), relying mostly on colleagues and personal networks. This lack of institutional support has previously been identified as a risk factor for burnout.^29^

The overall mental health impact may be broader, considering the lack of awareness among physicians about burnout, as previously reported in a qualitative study.^10^ Burnout impacts on patient safety and physician productivity.^28^^,^^30^ Respondents describe distress following medical error as being both psychological and cognitive, and this can have negative personal consequences commensurate with PTSD, as recently highlighted during the COVID-19 pandemic.^31^^,^^32^ Among surgical trainees, about 12% were reported to present with PTSD following stressful situations, and patient death has been shown to put both younger and more experienced surgeons under sufficient psychological stress, potentially causing burnout, depression, or substance abuse.^33^ This contrasts with intensive care unit physicians, for whom patient death was found to be unrelated to burnout in 1 study.^9^ These results are particularly sobering considering that the emotional impact of patient death on cardiologists remains ill-known and taboo.^33^^,^^34^ CPs are trained to save lives and report being ill-prepared in medical school to face death.^35^, ^36^, ^37^, ^38^, ^39^ Lastly, our findings show that CPs also face high work intensity, emotional demands, and conflict in the workplace. These are 3 of the 6 psychosocial risks in the workplace, which also include insufficient autonomy, and lack of a good social rapport in the workplace.

### Compensatory strategies

Among physicians reporting high emotional impact, a substantial proportion reported using maladaptive coping strategies. More than one-quarter of physicians reported the use of psychotropic medication, or substances, whereas only 12% (n = 24) sought psychological support. This level of health-seeking behavior is lower than that reported in a large survey of American cardiologists.^1^ Instead, physicians often remain silent about the emotional impact of patient death.^40^ Defensive coping strategies such as denial, anger, avoidance, or blaming others may be used.^28^^,^^34^ These patterns are consistent with the "second victim" phenomenon, whereby health care providers who experience the death or serious harm of a patient may suffer in silence due to professional culture and stigma.^31^ However, psychological support has been shown to be effective in preserving physician mental health. Recognizing and addressing psychological distress is therefore essential.^41^, ^42^, ^43^

### Clinical and Organizational implications for practice

The consequences of patient death extend beyond individual physicians to the health care system. Higher emotional impact was associated with an increased frequency of conflictual relationships within teams. These conflicts may reflect behavioral responses to stress and may impair team cohesion and communication. In addition, burnout and psychological distress can negatively affect decision-making, communication, and professional behavior.^44^ Institutional factors, including high workload and chronic understaffing, may further exacerbate these effects.^45^ Physicians in this study reported long working hours and prolonged periods without leave, consistent with known psychosocial risk factors.^45^^,^^46^

Burnout is associated with reduced career satisfaction, decreased productivity, absenteeism, and increased health care system costs.^47^, ^48^, ^49^ Thus, physician burnout has serious negative consequences to the health care system as a whole but also to future patients and families.^50^

Despite the high frequency of patient death in cardiac practice, its psychological toll on CPs remains a largely overlooked dimension of professional life. Growing evidence, including recent guidance from the European Society of Cardiology, calls for greater attention to physician mental health within cardiovascular medicine, yet the specific burden linked to patient loss continues to receive insufficient institutional recognition.^51^ Addressing this gap requires coordinated action at multiple levels: health care organizations and professional bodies must acknowledge the emotional weight of patient death as a dual threat to physician well-being and the quality of patient care.^28^ Building organizational resilience through reduced workload pressures, improved work-life balance, stronger peer networks, and psychologically safe working environments represents a necessary and actionable priority across individual, team, and institutional domains.^52^, ^53^, ^54^ Evidence from high-performing “magnet hospitals”—where professional autonomy, collaborative culture, and adequate staffing are systematically promoted—offers a concrete model for this kind of systemic change.^55^ Targeted programs delivered at the individual level, including structured debriefing sessions, peer support schemes, mentorship pathways, and training in emotional regulation skills, should become standard components of cardiac medicine education and continuing professional development.^56^ Situating these efforts within the “second victim” framework—which characterizes the psychological harm sustained by clinicians following patient deterioration or death—may help dismantle the culture of silence that surrounds emotional distress in medicine and promote earlier engagement with support services; formal recovery programs informed by this framework have demonstrated meaningful benefit in comparable settings.^57^^,^^58^ Going forward, the field would benefit from the development and psychometric validation of multidimensional instruments specifically designed to capture the emotional impact of patient death, as well as from longitudinal research capable of clarifying causal mechanisms and from rigorously designed trials evaluating the effectiveness of support interventions in CP populations.

### Study limitations

First, findings may not be generalizable to other health care systems or countries. Second, the overall response rate of 12.2% is a notable limitation. Although consistent with voluntary online surveys on sensitive topics in medical populations, selection bias cannot be excluded: physicians most affected may have been more likely to respond, whereas those most distressed may have lacked the capacity to do so. Findings should therefore be interpreted with caution and may not be representative of French CPs as a whole. The survey was conducted shortly after the COVID-19 pandemic, within the context of a strained health care system, which may have further influenced the results. Third, although depression, anxiety, PTSD, and burnout were assessed with validated screening tools, not diagnostic instruments, the EIPD scale was not formally validated. This is a key limitation: as a single-item measure, EIPD does not allow assessment of internal consistency or dimensional structure, and may be less robust than multi-item instruments. Future studies should develop and validate multidimensional tools to better characterize the emotional impact of patient death. Fourth, the HADS was originally developed for medically ill populations; findings should therefore be interpreted as indicative of symptoms rather than clinical diagnoses. Fifth, physicians with higher levels of burnout or depressive symptoms may perceive and report patient death differently, potentially introducing reporting bias. Sixth, the cross-sectional design precludes any inference of causality or temporal sequence between emotional impact and psychological distress outcomes. Seventh, subgroup analyses were limited by the exploratory nature of this study and the need to reduce type I error risk, which may partly explain differences observed between specialties.

## Conclusions

These findings demonstrate a significant association between the emotional impact of patient death and psychological distress among cardiovascular physicians, highlighting an important and under-recognized dimension of physician well-being. They underscore the need for improved recognition and the development of targeted support and training initiatives.

Perspectives **COMPETENCY IN PATIENT CARE AND MEDICAL KNOWLEDGE:** Patient death is a frequent and emotionally significant event for CPs, yet its psychological consequences remain largely unrecognized and unaddressed in clinical culture. This study demonstrates that a high emotional impact of patient death is independently associated with symptoms of anxiety, depression, PTSD, and burnout. Female gender, cardiothoracic surgical specialty, and a personal history of trauma are key risk factors. Physicians with high emotional impact are more likely to resort to maladaptive coping strategies, including psychotropic medication and addictive substances, while rarely seeking psychological support. Despite growing recognition of the importance of mental health in cardiovascular care, the psychological burden experienced by physicians themselves remains insufficiently addressed. These findings highlight the need for CPs to develop awareness of their own emotional responses to patient death as a core component of professional competence and self-care.

**TRANSLATIONAL OUTLOOK:** Health care institutions and professional societies should recognize the emotional burden of patient death as both a physician well-being and patient safety issue. Organizational resilience, encompassing improvements in workload, work-life balance, social support, and the creation of psychologically safe team environments, should be fostered at individual, team, and institutional levels. The conditions associated with high-performing “magnet hospitals,” including professional autonomy, collaborative teamwork, and adequate staffing, provide a useful framework for institutional action. At the individual level, structured interventions such as psychological debriefing, peer support programs, mentorship, and training in emotional regulation should be integrated into cardiac medicine training and practice. Framing this burden within the established “second victim” framework may further help reduce institutional stigma and encourage help-seeking; structured recovery support programs have been shown to facilitate this process. Future research should prioritize the validation of multidimensional tools to assess the emotional impact of patient death, longitudinal studies to establish causal pathways, and rigorous evaluation of targeted support programs in CP populations.

## Funding support and author disclosures

This study received institutional grant from the Association pour la Recherche Multidisciplinaire en Cardiologie. The authors have reported that they have no relationships relevant to the contents of this paper to disclose.

## Supplementary material

Supplementary pdf 1 Supplementary Figure S1. Mean EIPD Scores by Psychological Distress Symptom Level Mean Emotional Impact of Patient Death (EIPD) scores according to the presence of low or high levels of psychological distress: PTSD-related symptoms (IES-R), anxiety (HADS), depression (HADS), and burnout (MBI). Bars represent mean EIPD scores ± SD. P values were calculated using independent samples t-test. EIPD scores were significantly higher among physicians with PTSD-related symptoms (p = 0.004) and severe burnout (p = 0.002), whereas no significant difference was observed for anxiety or depression (p = 0.068 and p = 0.275, respectively). These analyses are presented for descriptive purposes given the cross-sectional design. HADS = Hospital Anxiety and Depression Scale; IES-R = Impact of Event Scale-Revised; MBI = Maslach Burnout Inventory; NS = not significant.
