# Supplementary material for: Emotional Impact of Patient Death and Associated Psychological Distress in Cardiac Physicians
Source: JACC Adv. 2026 Jun 17;5(7):102861. doi: 10.1016/j.jacadv.2026.102861 (PMC13311153; doi:10.1016/j.jacadv.2026.102861)
Supplement: Supplementary pdf 1 — Supplementary Figure S1. Mean EIPD Scores by Psychological Distress Symptom Level Mean Emotional Impact of Patient Death (EIPD) scores according to the presence of low or high levels of psychological distress: PTSD-related symptoms (IES-R), anxiety (HADS), depression (HADS), and burnout (MBI). Bars represent mean EIPD scores ± SD. P values were calculated using independent samples t-test. EIPD scores were significantly higher among physicians with PTSD-related symptoms (p = 0.004) and severe burnout (p = 0.002), whereas no significant difference was observed for anxiety or depression (p = 0.068 and p = 0.275, respectively). These analyses are presented for descriptive purposes given the cross-sectional design. HADS = Hospital Anxiety and Depression Scale; IES-R = Impact of Event Scale-Revised; MBI = Maslach Burnout Inventory; NS = not significant. [file mmc1.pdf]

Supplemental Appendix

1. Supplementary Figure S1: Mean Emotional Impact of Patient Death (EIPD) scores according to the presence of low level or high level of psychological distress symptoms: PTSD-related symptoms (IES-R), symptoms of anxiety (HADS), symptoms of depression (HADS) and burnout (MBI).

Bars represent mean EIPD scores ± standard deviation. P-values were calculated using independent samples t-test. EIPD scores were significantly higher among physicians with PTSD-related symptoms (p=0.004) and severe burnout (p=0.002), whereas no significant difference was observed for anxiety and depression symptoms (p=0.068 and p=0.275, respectively). These analyses are presented for descriptive purposes given the cross-sectional design. EIPD = Emotional Impact of Patient Death; HADS = Hospital Anxiety and Depression Scale; IES-R = Impact of Event Scale-Revised; MBI = Maslach Burnout Inventory; NS = not significant; SD = standard deviation.

2. Supplementary Table S1. Sensitivity Analysis: Linear Regression with EIPD as a Continuous Outcome

Caption: Each unit increase in EIPD score is associated with a 0.787-point increase for female gender, a 0.146-point increase per unit of personal trauma history, a 1.899-point increase for cardiothoracic surgeons, a 2.050-point increase for cardio-pediatrician, and a 0.920-point increase for cardiologists, all compared to anesthesiologists. These findings are consistent with the primary logistic regression analysis (Table 5).

Model fit: $R = 0.339$; $R^2 = 0.115$; Adjusted $R^2 = 0.109$; Std. Error of estimate = 1.900; $F(5, 736) = 19.050$, $p < .001$; N = 742; Durbin-Watson = 1.900; B = unstandardized regression coefficient; SE = standard error; CI = confidence interval; [a] Reference category: cardiovascular anesthesiologist; ** $p < .01$; *** $p < .001$.

3. Supplementary Table S2: Demographic, occupational, and psychological characteristics of cardiothoracic surgeons compared with non-surgeon cardiac physicians.

Caption: Interpretation: This analysis is exploratory and presented for descriptive purposes; given the small sample size of the surgeon subgroup (n=62), results should be interpreted with caution. Notably, cardiothoracic surgeons reported higher EIPD scores despite experiencing fewer patient deaths per year than non-surgeon physicians, suggesting that the nature and perceived responsibility of death exposure — rather than its frequency — may be the primary determinant of emotional impact in this subgroup.

EIPD = Emotional Impact of Patient Death; IES-R = Impact of Event Scale-Revised; MBI = Maslach Burnout Inventory; HADS = Hospital Anxiety and Depression Scale; IQR = interquartile range; SD = standard deviation. Data are expressed as mean ± SD or n (%) unless otherwise stated. P-values were calculated using independent samples t-test for continuous variables and chi-square test for categorical variables.

4. **List of French Cardiac Physicians that participated in the study**
5. **Questionnaire translated in English**

Supplementary Figure S1. Mean Emotional Impact of Patient Death (EIPD) scores according to the presence of low level or high level of psychological distress symptoms: PTSD-related symptoms (IES-R), symptoms of anxiety (HADS), symptoms of depression (HADS) and burnout (MBI).

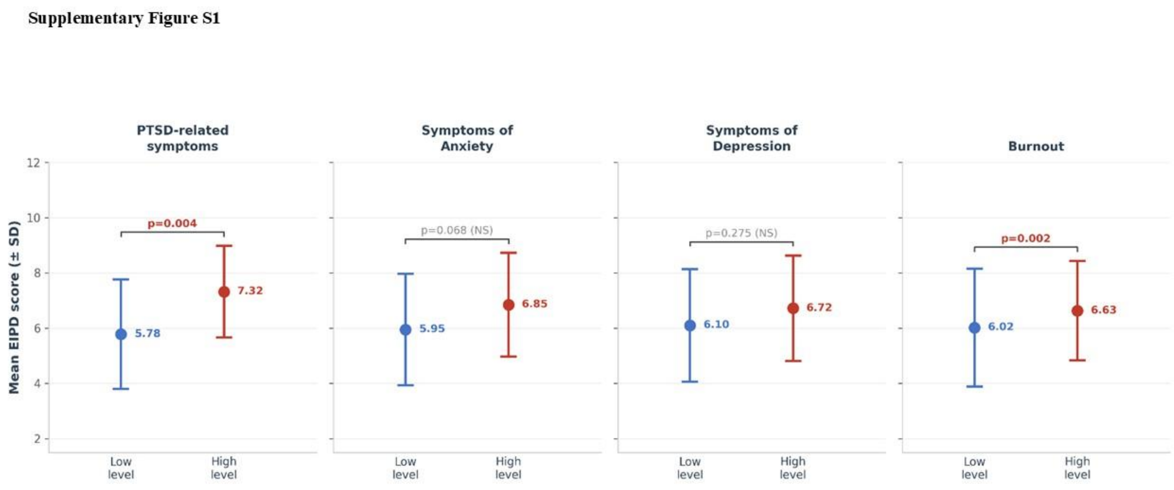

Supplementary Table S1. Sensitivity Analysis: Linear Regression with EIPD as a Continuous Outcome

Variable	B	SE	t	p	95% CI	Sig.
Constant	6.763	0.317	21.339	<.001	[6.141 ; 7.384]	***
Female gender	0.787	0.143	5.488	<.001	[0.506 ; 1.068]	***
**Personnal Post Traumatic Disorders** (1–10)	0.146	0.024	6.098	<.001	[0.099 ; 0.193]	***
Cardiothoracic surgeon [a]	**1.899**	0.390	4.873	<.001	[1.135 ; 2.664]	***
Cardio-pediatrician [a]	2.050	0.624	3.287	.001	[0.828 ; 3.272]	**
Cardiologist [a]	0.920	0.212	4.330	<.001	[0.503 ; 1.336]	***

Supplementary Table S2: Demographic, occupational, and psychological characteristics of cardiothoracic surgeons compared with non-surgeon cardiac physicians.

Cardiac physicians<br>N	Surgeons<br>62	Non surgeons<br>685	p
**Demographic variables**			
Age. years	43.73 ± 11.81	44.56 ± 12.37	0.23
Females, n (%)	11 (17.7)	311 (45.7)	0.0001
Do you have a traumatic history (death, accident, aggression)? 1 to 10	3.08 ± 2.74	3.60±1.73	0.100
**Characteristic of the work**			
Number of years' experience	18.39 ± 14.59	19.40 ± 15.00	0.734
Hours worked per week	71.6 ± 69.0	53.5 ± 12.21	0.0001
Weekend on-call shifts per month	8.05 ± 4.15	3.38 ±4.80	0.009
Quality of relationship with Institution, 1(very bad) to 10 (excellent)	5.03 ± 2.43	5.02 ± 2.21	0.229
Quality of relationship with healthcare co-workers, 1(very bad) to 10(excellent)	8.10 ± 1.50	7.90 ± 1.46	0.758
Conflictual relationship with Co-workers, Yes vs No or not applicable	22 (35.5)	204 (29.8)	0.349
**Emotional Impact of Patient's Death (EIPD)**			
Number of your patients who died in the last year? Median (IQR) and Mean ±SD	6.89 ± 7.25	17.14 ±23.26	0.001
EIPD, mean (sd)	6.90 ± 1.57	6.24 ± 2.04	0.017
EIPD, % Q3-Q4 vs Q1-Q2	42 (67.7)	358 (52.3)	0.019
Could you estimate your emotional impact when faced with…			
Death occurring after a long follow-up	6,27 ± 2.61	7.02 ± 2.27	0.058
When you identify with the patient	5.08 ± 2.77	5.90 ± 2.78	0.514
Death of a young patient	7.94 ± 2.30	8.09 ± 1.88	0.219
Death due to a cardiovascular cause	5.52 ± 2.92	5.09 ± 2.88	0.954
Unexpected death (sudden)	7.85 ± 1.88	7.54 ± 2.07	0.294
Death occurring during an intervention	8.05 ± 2.36	7.90 ± 2.22	0.572
Death occurring after an intervention	7.66 ± 1.62	7.34 ± 2.12	0.071
**Following the death of your patients did you…**			
Take psychotropic drug(s)			0.384
Yes	1 (1.6)	34 (5.0)	
No	61 (98.4)	646 (94.3)	
I prefer not to reply	**0 (0)**	5 (0.7)	
Consult a psychologist or psychiatrist,			0.094
Yes	**0 (0)**	35 (5.1)	
No	61 (98.4)	647 (94.5)	
I prefer not to reply	1 (1.6)	3 (0.4)	
Take substances (alcohol, cannabis)			0.816
Yes	8 (12.9)	81 (11.8)	
No	53 (85.5)	598 (87.3)	
I prefer not to reply	1 (1.6)	6 (0.9)	
**Do you feel helped after the death of one of your patients?**			
By your colleagues, 1 to 10	5.58 ± 2.96	5.54 2.797	0.493
By your institution, 1 to 10	1.74 ± 2.59	1.46 ± 2.13	0.049

By your family or friends, 1 to 10	6.84 ± 2.94	5.24 ± 3.07	0.137
**PTSD-related symptoms (IESR)**	25 (40.3)	223 (32.6)	0.214
Intrusion	10.5 (6.0; 16.0)	8.0 (4.0; 14.0)	0.006
Avoidance	7 (2.0; 11.3)	5.0 (2.0; 10.0)	0.232
Hyperarousal	3.5 (1.0; 7.0)	3.0 (1.0; 6.0)	0.530
**Symptoms of anxiety**	18 (29)	264 (38.5)	0.139
**Symptoms of Depression**	21 (33.9)	211 (30.8)	0.617
**Symptoms of severe burnout, n (%) vs medium and low burnout (MBI)**	30 (48.4)	305 (44.5)	0.558
Exhaustion	18.0 (10.8; 30.0)	20 (12.0; 29.0)	0.284
Depersonalization	6.5 (2.0: 13.0)	7.0 (4.0; 12.5)	0.712
Personal achievement	40.0 (34.8; 44.0)	40.0 (35.0; 44.0)	0.955

List of French Cardiac Physicians that participated in the study:

Dr Muriel BIGOT, president of the Groupe Exercice Réadaptation et Sport (GERS) of the Société Française de Cardiologie (SFC); Dr Guillaume BONNET, president of the Collège des Cardiologues en Formation (CCF) of SFC; Prof Guillaume CAYLA, president of Groupe Athérome Coronaire et Interventionnelle (GACI) of SFC; Prof Ariel COHEN, president of the SFC; Dr Serge COHEN, président of the Collège Nationale des Cardiologues Français (CNCF); Madame Armelle DUCHENNE, Head of Nursing, Henri Mondor Hospital; Dr Stéphane EDHERY, president of Cerce Cardio-Oncologie of SFC; Prof Laurent FAUCHIER, president of Groupe Rythmologie - Stimulation Cardiaque of SFC; Dr Albert FRANCK, president of the Collège Nationale des Cardiologues Hospitaliers (CNCH) of SFC; Madame Mounira KHAROUBI, Ingénieur de Recherche, Hopital Henri Mondor; Prof Nicolas MANSENCAL, president of Filiale d'Imagerie Cardiovasculaire; Madame Soizic MASSARD, Head of Nursing, CHU Rennes Pontchaillou; Dr Nicolas LAMBLIN, president of Groupe Insuffisance Cardiaque et Cardiomyopathies (GICC) de la SFC; Prof Christophe LECLERC, vice president of SFC; Prof Atul PATHAK, president of Société française d’hypetension artierielle (SFHTA); Dr Théo PEZEL, vice-president of CCF; Madame Julie POMPOUGNAC, psychologist, Henri Mondor Hospital; Prof Etienne PUYMIRA, president of the Groupe UISC of SFC; Madame Agathe RAUWEL, Secrétaire médicale, Hôpital Henri Mondor, Créteil; Madame Agnès ROCHE, Head of Nursing, CHU Pontchaillou, Rennes; Dr Pierre SABOURET, secretary of CNCF; Prof Jean-Benoit THAMBO, president of Filiale de Cardiologie Pédiatrique et Congénitale of SFC; Madame Véronique THORÉ, Head of Nursing, CHRU Nancy, Nancy; Madame Stéphanie TURPEAU, Head of Nursing, president of the Groupe Paramédicaux of SFC; Prof Olaf MERCIER, president of the Conseil Scientifique de la SFCTCV; Madame Anne SPINOSI, administratrice de la SFCTCV; Soizic MASSARD, Cadre de santé CHU Rennes.

Questionnaire translated in English

PART I-ABOUT YOU

A-REGARDING YOUR WORK:

-What is your specialty?
□ Cardiac Surgery Anesthesiologist-Intensivist □ Cardiologist □ Cardiac Surgeon
-What is your main area of activity (one answer only)?
□ Cardiac Surgery Anesthesia-Intensive Care □ General Cardiology □ Intensive Coronary Care Unit-ICU □ Medical Valvular Cardiology □ Pediatric and Congenital Cardiology □ Heart Failure and Cardiomyopathy □ Interventional Cardiology □ Electrophysiology □ Cardiological Imaging □ Cardiac Rehabilitation □ Cardiac Surgery (excluding transplant and assist programs) □ Cardiac Surgery with Transplant/Transplantation/Assistance
-What is your status?
□ Resident □ Fellow / Assistant □ Senior Physician (Private Practice, Hospital Practitioner) □ University Faculty (Associate Professor or Professor)
-How many years have you been practicing (since the start of your residency)?
_ _ years
-What is your primary place of practice?
□ Private Practice □ General Hospital □ University Hospital □ Clinic □ Mixed (Hospital/Private Practice) □ Mixed (Clinic/Private Practice)
-In which region is your main place of practice located? (13 regions offered in a drop-down menu)

B-REGARDING YOUR PERSONAL LIFE:

-What is your age? _ _ years:
-You are:
□ Male □ Female
-You live:
□ With a partner □ With family □ Alone

C-REGARDING YOUR QUALITY OF LIFE AT WORK:

-What is your average daily commute time (round trip)? : _ _ _ minutes
-What is your average weekly working time (clinical + administrative)?: _ _ _ hours
-Number of on-call or half-on-call duties per month:
□ None □ 1 □ 2 □ 3 □ 4 □ 5 □ 6 □ 7 □ 8 □ 9 □ 10 or more
-Number of on-call duties per month (including weekends):
□ None □ 1 □ 2 □ 3 □ 4 □ 5 □ 6 □ 7 □ 8 □ 9 □ 10 or more
1-How would you rate your relationship with the administration (Hospital, Institution, Health Insurance)?
□ 1 (Very Poor) □ 2 □ 3 □ 4 □ 5 □ 6 □ 7 □ 8 □ 9 □ 10 (Excellent) □ Not applicable
-How would you rate your relationship with the healthcare professionals you work with daily?
□ 1 (Very Poor) □ 2 □ 3 □ 4 □ 5 □ 6 □ 7 □ 8 □ 9 □ 10 (Excellent) □ Not applicable
-Are you in conflict with at least one person in your department or practice?
□ Yes □ No □ Not applicable
15-Do you consider yourself adequately financially compensated for your work?
□ 1 (Insufficiently) □ 2 □ 3 □ 4 □ 5 □ 6 □ 7 □ 8 □ 9 □ 10 (Adequately)
16-How long has it been since you had a full week of vacation without working (no work, no emails, no professional opinions)?
_ _ _ _ _ _ days

D-YOUR PATIENTS:

-How would you rate the quality of your relationships with patients?
□ 1 (Very Poor) □ 2 □ 3 □ 4 □ 5 □ 6 □ 7 □ 8 □ 9 □ 10 (Excellent)
-How would you rate the quality of your relationships with patients' families?
□ 1 (Very Poor) □ 2 □ 3 □ 4 □ 5 □ 6 □ 7 □ 8 □ 9 □ 10 (Excellent)

PART II ABOUT DEATH

A-THE DEATH OF YOUR PATIENTS

-How many of your patients have died in the past year (anywhere)? : _ _ _ number
-How many of your patients have died in the past month (anywhere)? : _ _ _ number
-What percentage of patient deaths in the past year were sudden (unexpected)?: _ _ _ % (1 to 100)
-In the past month, have you made or participated in a decision to stop or limit treatment? If so, how many:
□ None □ 1 □ 2 □ 3 □ 4 □ 5 □ 6 □ 7 □ 8 □ 9 □ ≥10

B-THE DEATH OF YOUR PATIENTS – FOR YOU

-Do you think the deaths of your patients have an emotional/affective impact on you?
□ 1 (None); □ 2; □ 3; □ 4; □ 5; □ 6; □ 7; □ 8; □ 9; □ 10 (Major)
-How would you assess the emotional impact of the following factors ON YOU during the death of one of your patients?

- Length of follow-up before death:
  □ 1 (None); □ 2; □ 3; □ 4; □ 5; □ 6; □ 7; □ 8; □ 9; □ 10 (Major)
- Your identification with the patient:
  □ 1 (None); □ 2; □ 3; □ 4; □ 5; □ 6; □ 7; □ 8; □ 9; □ 10 (Major)
- Young age of your patient:
  □ 1 (None); □ 2; □ 3; □ 4; □ 5; □ 6; □ 7; □ 8; □ 9; □ 10 (Major)
- Cardiovascular cause of death:
  □ 1 (None); □ 2; □ 3; □ 4; □ 5; □ 6; □ 7; □ 8; □ 9; □ 10 (Major)
- Sudden (unexpected) nature of death:
  □ 1 (None); □ 2; □ 3; □ 4; □ 5; □ 6; □ 7; □ 8; □ 9; □ 10 (Major)
- Death occurring during a procedure (prescription/act/surgery):
  □ 1 (None); □ 2; □ 3; □ 4; □ 5; □ 6; □ 7; □ 8; □ 9; □ 10 (Major); □ Not applicable
- Death occurring after a procedure (prescription/act/surgery):
  □ 1 (None); □ 2; □ 3; □ 4; □ 5; □ 6; □ 7; □ 8; □ 9; □ 10 (Major)

-Have the deaths of your patients led you to:

- Take psychotropic medication: Yes; No
- Consult a specialist: Yes; No
- Consume substances (alcohol, cannabis, etc.): Yes; No
- Take a break from your professional activities: Yes; No

C-ABOUT YOURSELF

-Do you have personal traumatic experiences (bereavement, accident, assault, etc.)?
□ 1 (None); □ 2; □ 3; □ 4; □ 5; □ 6; □ 7; □ 8; □ 9; □ 10 (A lot)
-How would you rate the quality of your relationships with close family or your social circle?
□ 0 (Very Poor) □ 1; □ 2; □ 3; □ 4; □ 5; □ 6; □ 7; □ 8; □ 9; □ 10 (Excellent)
-Do you practice regular physical activity (i.e., at least weekly)?: □ Yes; □ No
-Do you practice yoga, meditation, or sophrology?: □ Yes; □ No; □ I prefer not to answer
-Are you a believer?: □ Yes; □ No; □ I am questioning; □ I prefer not to answer
-Do you have a religious practice?: □ Yes; □ No; □ I prefer not to answer

E-MODALITIES OF HANDLING DEATH

-Do you feel supported when a patient of yours dies?

- By your colleagues, team, and/or department:
  □ 1 (Not at all); □ 2; □ 3; □ 4; □ 5; □ 6; □ 7; □ 8; □ 9; □ 10 (Very much)
- By the institution:
  □ 1 (Not at all); □ 2; □ 3; □ 4; □ 5; □ 6; □ 7; □ 8; □ 9; □ 10 (Very much)
- By your family or social circle:
  □ 1 (Not at all); □ 2; □ 3; □ 4; □ 5; □ 6; □ 7; □ 8; □ 9; □ 10 (Very much)

-Do you think end-of-life care is sufficiently taught in medical education? :Yes; No

F-AND THE COVID-19 PANDEMIC

-How many symptomatic COVID-19 patients have you managed in the acute (infectious) and/or chronic (post-COVID-19 syndrome) phases?

□ None; □ <5; □ 6-10; □ 11-15; □ 21-30; □ More than 30; □ Not applicable

PART-III OTHER QUESTIONNAIRES

A-HADS

B-MDI

C-IESR
